# *tidyMicro*: a pipeline for microbiome data analysis and visualization using the *tidyverse* in R

**DOI:** 10.1186/s12859-021-03967-2

**Published:** 2021-02-01

**Authors:** Charlie M. Carpenter, Daniel N. Frank, Kayla Williamson, Jaron Arbet, Brandie D. Wagner, Katerina Kechris, Miranda E. Kroehl

**Affiliations:** 1 Department of Biostatistics and Informatics, Colorado School of Public Health, University of Colorado Anschutz Medical Campus, Aurora, CO USA; 2 Division of Infectious Diseases, Department of Medicine, University of Colorado Anschutz Medical Campus, Denver, CO USA

**Keywords:** Microbiome, Pipeline, R, Tidyverse, Visualization, Open source

## Abstract

**Background:**

The drive to understand how microbial communities interact with their environments has inspired innovations across many fields. The data generated from sequence-based analyses of microbial communities typically are of high dimensionality and can involve multiple data tables consisting of taxonomic or functional gene/pathway counts. Merging multiple high dimensional tables with study-related metadata can be challenging. Existing microbiome pipelines available in R have created their own data structures to manage this problem. However, these data structures may be unfamiliar to analysts new to microbiome data or R and do not allow for deviations from internal workflows. Existing analysis tools also focus primarily on community-level analyses and exploratory visualizations, as opposed to analyses of individual taxa.

**Results:**

We developed the R package “*tidyMicro”* to serve as a more complete microbiome analysis pipeline. This open source software provides all of the essential tools available in other popular packages (e.g., management of sequence count tables, standard exploratory visualizations, and diversity inference tools) supplemented with multiple options for regression modelling (e.g., negative binomial, beta binomial, and/or rank based testing) and novel visualizations to improve interpretability (e.g., Rocky Mountain plots, longitudinal ordination plots). This comprehensive pipeline for microbiome analysis also maintains data structures familiar to R users to improve analysts’ control over workflow. A complete vignette is provided to aid new users in analysis workflow.

**Conclusions:**

*tidyMicro* provides a reliable alternative to popular microbiome analysis packages in R. We provide standard tools as well as novel extensions on standard analyses to improve interpretability results while maintaining object malleability to encourage open source collaboration. The simple examples and full workflow from the package are reproducible and applicable to external data sets.

## Background

The microbiome plays a large, sometimes even causal, role in the conditions of their environment [1–5]. The drive to understand the interplay between microbiota community structure and host pathophysiology has sparked active research across several fields. Technological advances in sequencing accuracy, depth, and throughput now allow comprehensive analysis of microbial communities through both targeted (i.e., marker-gene surveys, such as 16S rRNA genes) and untargeted (i.e., shotgun metagenomics) approaches. However, the high dimensional nature of these data can be difficult to manage. Research projects often involve management and integration of multiple sequence-based datasets, along with the corresponding clinical data. Merging these data sets together into a workable format can be difficult and time consuming.

This problem inspired microbiome analysis pipelines to construct their own data structures for data management, the most popular in R [6] being the *phyloseq*-class [7, 8]. The majority of omics analysis pipelines available in R rely on “S4” level objects since they provide flexibility beyond data frames and lists. However, these data structures impose severe limitations to the users’ control over their analysis and do not accommodate functions from external packages. Furthermore, popular microbiome pipelines focus heavily on community level analyses and exploratory visualizations (e.g. ordination plots, stacked bar charts of raw abundances). Those who offer taxon level models do not provide model visualization techniques. Model visualizations tailored to microbiome data would improve collaboration and convey “big picture” results better than using large summary tables alone.

We introduce a new R package, “*tidyMicro*,” that extends typical components of microbiome analysis tools by incorporating several new data analysis and visualization tools while following the principles of the *tidyverse *[9]. These data structures and workflow principles grant the capability to adopt user extensions and integrate external functions, a unique feature that distinguishes *tidyMicro* from other R-based microbiome analysis pipelines. The viability of the pipeline was evaluated using published sequencing data from a study on bacterial community relationships to bronchopulmonary dysplasia in preterm infants [10] and data from a study on the nasal microbiome of hospital inpatients suffering from Staphylococcus aureus infections [11]. The package is currently available through GitHub and CRAN.

## Implementation

**Fig. 1:**
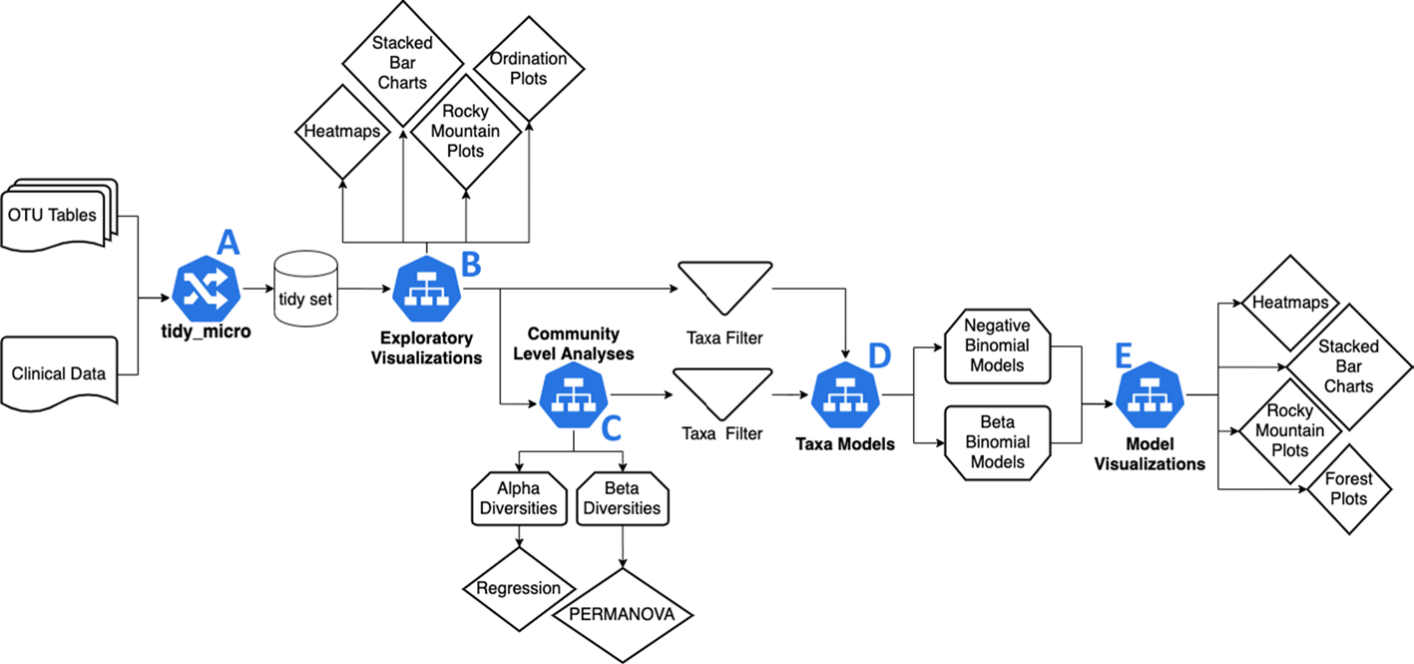
Flowchart of the *tidyMicro* pipeline. The supplied OTU table(s) must be in the standard format output by QIIME with column names that match a sequencing library names column in the clinical data. The initial step is merging all OTU tables and clinical data using the *tidy_micro* function (**a**). From here, the *tidyMicro* set can be used for exploratory visuals (**b**), community level analyses (**c**), and taxa level analyses (**d**, **e**)

The *tidyMicro* pipeline consists of 5 macro operations with several options within each (Fig. 1). All operations are implemented and supported through R, with graphics and coding style following the general style of the *tidyverse*. The first step in the pipeline is merging OTU table(s) and clinical data together into a “tidy” format (Fig. 1a). From here, several functions are provided to explore the data visually (Fig. 1b), calculate and analyze community diversity measures (i.e. alpha-diversity and beta-diversity analyses; Fig. 1c), model abundance and prevalence of individual taxa (Fig. 1d), and summarize and visualize model results (Fig. 1e).

In this paper, we demonstrate a standard workflow using two published data sets. We use data from a study that set out to define the nasal microbiome of hospital inpatients who are persistently colonized with methicillin-resistant Staphylococcus aureus (MRSA) compared with matched, non-colonized controls [11] as our primary example set. We also use a subset of a published data set from a study assessing connections between airway taxa and bronchopulmonary dysplasia (BPD) severity in prematurely born infants [10]. The BPD study allows us to demonstrate a new visualization technique for longitudinal data and the package’s ability to handle multiple OTU tables. These examples do not illustrate all possible options and functions available through *tidyMicro*. Instead they serve as a useful overview of the pipeline’s primary functions with full descriptions of novel features. It also demonstrates the pipeline’s ability to create reproducible microbiome analyses through popular open source software.

### Motivating examples

#### MRSA study

In the MRSA study researchers acquired samples from an active MRSA screening from the Department of Veterans Affairs Eastern Colorado Health Care System. Patients were screened for nasal MRSA colonization at admission, inter-ward transfer, weekly intervals of a $\ge 7$ day stay in any ward, discharge, and death. An MRSA persistent carrier case was defined as someone from whom at least 5 swabs had been taken, at least 1 week apart, and $\ge 80\%$ of nasal swabs were positive for MRSA. An MRSA noncarrier control was defined as a someone from whom at least 5 swabs had been taken, at least 1 week apart, and all swabs were negative for MRSA. Noncarrier controls were then matched to persistent carriers based on known colonization risk factors including age, diabetes mellitus, long-term care residence (nursing home), and several others. Bacterial profiles were determined by broad-range amplification and sequence analysis of 16S ribosomal RNA (rRNA) genes. We have classified the sequencing reads into their genus level taxonomic ranks. This cohort’s demographics are summarized in Table 1.

#### Bronchopulmonary dysplasia

In the BPD study, tracheal aspirate samples were collected at 7, 14, and 21 days of life (± 48 h) from pre-term infants requiring mechanical ventilation. This subset of the original data set contains sequences from 24 infants, all ventilated at 7 days of life. Only 15 of these 24 had samples from all 3 time points. Bacterial profiles were determined by broad-range amplification and sequence analysis of 16S rRNA genes. We have classified the sequencing reads into their phylum, class, order, and family level taxonomic ranks to demonstrate the pipeline’s ability to manage multiple OTU tables. These cohort demographics are summarized in Table 2.

## Results:

### Data Structure

In a typical OTU table format [12–14], the first column contains OTU names and the following columns contain sequence read counts for each sample (sequencing library). The *tidy_micro* function can read in any number of OTU tables from targeted or untargeted sequencing methods and merge them with clinical data into a tidy data format. Merging four OTU tables from sequences classified to the phylum, class, order, and family level taxonomic ranks is demonstrated below:

$$tidy\_micro\left( {otu\_tabs = list\left( {Phylum = bpd\_phy,Class = bpd\_cla,Order = bpd\_ord,Family = bpd\_fam} \right),clinical = bpd\_clin,complete\_clin = TRUE} \right).$$

**Fig. 2:**
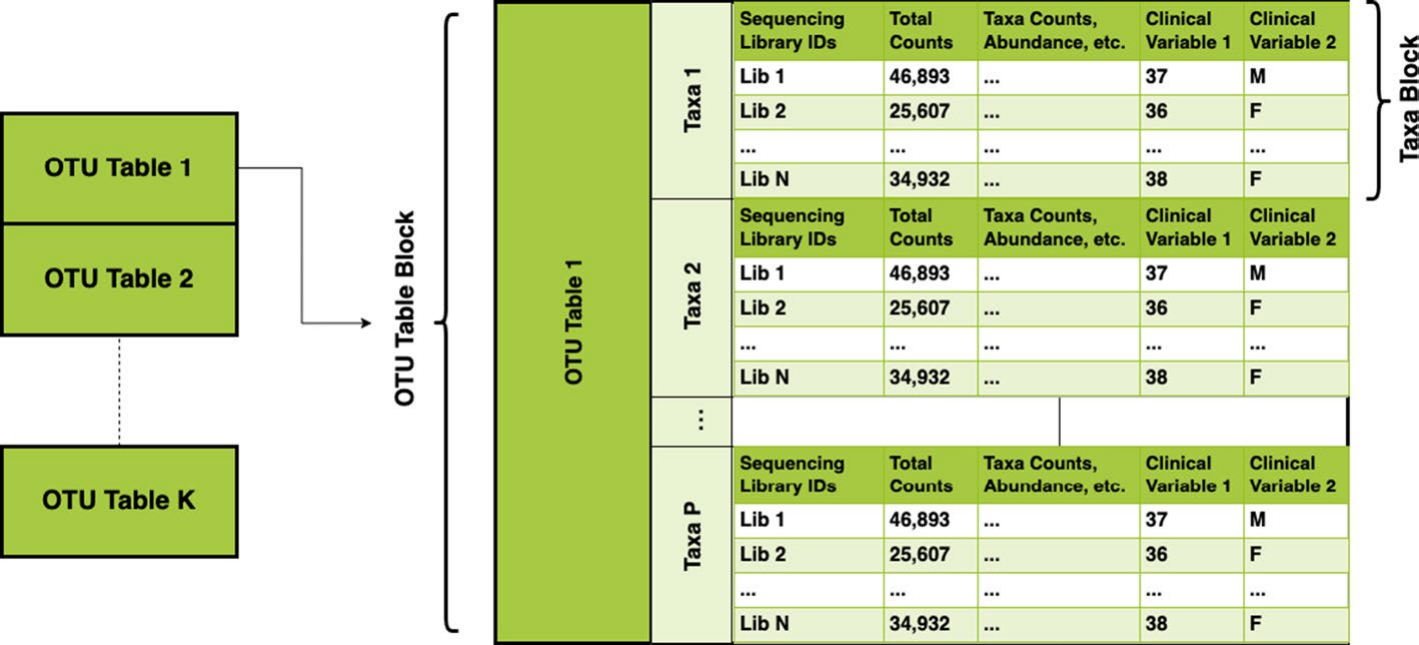
Structure of a *tidyMicro* data set. A *tidyMicro* data set is a data frame with a hierarchical structure where each OTU table creates a block containing taxa blocks for each taxa within the table. Clinical data is repeated within each taxa block. This structure allows users to easily create custom extensions

This creates a data frame with a hierarchical structure with a “block” for each OTU table and taxa “blocks” within each OTU table block (Fig. 2). The taxa blocks will contain the taxa counts for each sample and the samples’ corresponding clinical information.

Within each taxa block *tidy_micro* also calculates taxa presence/absence, relative abundance, and centered log ratio (CLR) transformed counts ${Z}_{p}=\mathrm{log}\left(\frac{{X}_{p}}{g\left({X}_{p}\right)}\right),$ where $g\left({X}_{p}\right)={\left({\prod }_{p}{X}_{p}\right)}^{1/P}$, ${X}_{p}$ is the observed count of taxa $p$, and $P$ is the number of taxa in the OTU table. Aitchison introduced the CLR as a useful transformation of compositional data to a Euclidean space [15]. We add $\frac{1}{sequencing\;depth}$ to each taxa’s count before the CLR transformation in order to avoid issues with $\mathrm{log}(0)$. Once data are wrangled into this framework the high dimensional nature of the data becomes manageable.

### Object Flexibility

The *tidyMicro* data format complies to the three primary principles of tidy data [16]: 1. each variable forms a column, 2. each observation forms a row, and 3. each type of observational unit forms a table. This malleable data structure allows users to easily work with familiar packages and functions outside of this pipeline. For example, creating a subset of a *tidyMicro* set is easily accomplished using standard operations within R such as *subset* or *filter*. New functions necessary for unique analyses can easily be applied within the OTU table or taxa blocks through *apply* functions or the *tidyverse*’s *d_ply* family. Similarly, all visualizations are created using *ggplot2,* meaning they can be easily customized through additional “geoms”. This ability to integrate base and external functions is unique among microbiome analysis pipelines. This flexibility will be key in fostering innovation from future users and encouraging the collaborative nature of open source software.

### Exploratory data analyses

**Fig. 3:**
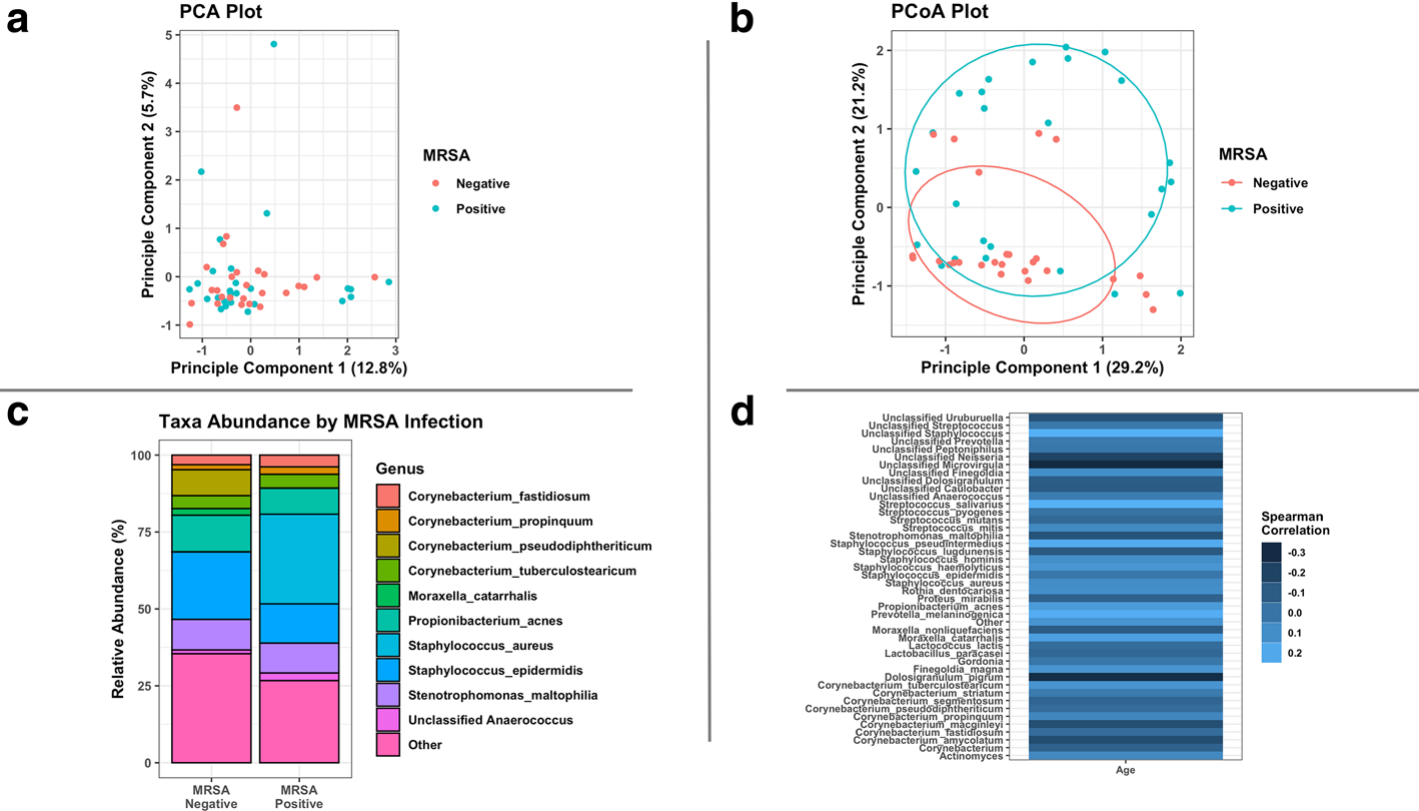
Example exploratory visualizations. **a** Principle component plot calculated from centered log ratio transformed genus level taxa counts, colored by MSRA infection. **b** Principle coordinate plot calculated from genus level Bray–Curtis beta diversity with normal ellipses, colored by MSRA infection. **c** Stacked bar charts of average genus level taxa abundances by MSRA infection. **d** Heatmap of Spearman correlations between centered log ratio transformed genus level taxa counts and subjects’ age

**Fig. 4:**
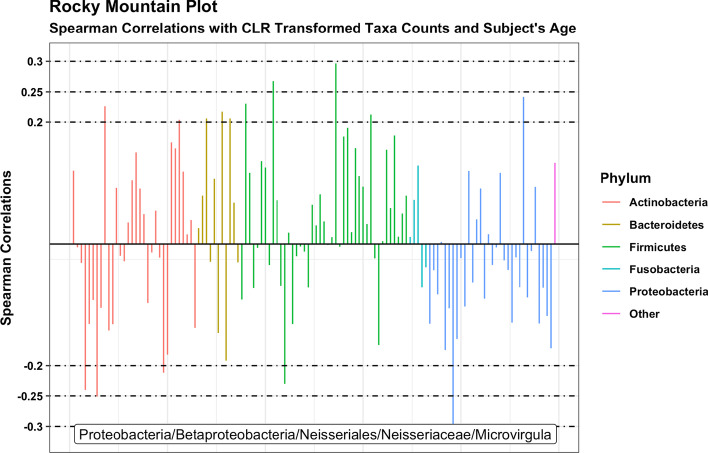
Rocky mountain plot. Spearman correlations between centered log ratio transformed genus level taxa counts and subjects’ age. Correlations are colored by phylum and taxa with correlations above 0.3 in magnitude are labeled

**Fig. 5:**
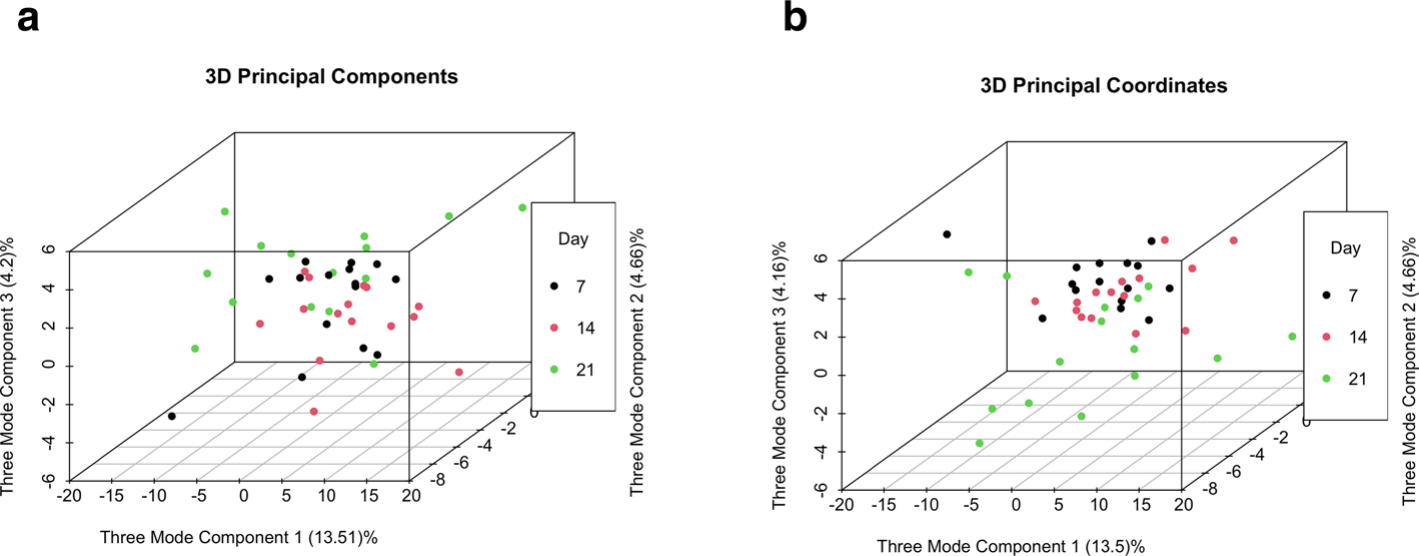
Three mode principle component (**a**) and three mode principle coordinate (**b**) plots. Plots created from sequences on the 7th, 14th, and 21st day of life of 15 infants collapsing over time component. Colors represent the three different time points. Principle coordinate plot created from Bray–Curtis beta diversity

Preliminary data exploration is straightforward with a *tidyMicro* set. We provide several wrapper functions for basic summary statistics of taxa abundance. Tabular outputs are complemented by graphical representations of abundance data, which can be much easier to interpret. We provide several standard data visualization tools including ordination plots (Fig. 3a, b), stacked bar charts (Fig. 3c), and heatmaps (Fig. 3d), as well as two novel visualization methods. The Rocky Mountain plot (Fig. 4) is a variant of the Manhattan plot that displays both the direction and magnitude of correlations between a variable of interest and each taxon within an OTU table, resembling the peaks and valleys seen along the front range of the Rocky Mountains. All taxa are colored by phylum, and correlations that exceed a desired magnitude will be automatically labeled using *ggrepel *[17]. Neither the CLR transformed counts nor the taxa abundances are normally distributed, so a rank-based correlation (e.g. Spearman, Kendall) is recommended and provided in the package. Three Mode principal component and principle coordinate analyses are tools used to account for the correlation structures of repeated measures within the ordination framework [18]. They collapse over the time component of variation and perform principle component / coordinate analyses on the remaining two dimensions (subject and taxa). For the motivating example, we display plots (using the 15 infants with all 3 sequencing time points) made using principle components (Fig. 5a) and principle coordinates (Fig. 5b).

### Taxa filtering

This package provides taxa “filters”. Counts from taxa below a specified prevalence and/or abundance cutoff will be aggregated into an “Other” taxa category. There is also an option to specify taxa that you are not interested in analyzing to be aggregated into this “Other” taxa. This is analogous to the “pruning” function in *phyloseq*. Filtering can be applied within the initial *tidy_micro* step or externally at a later stage. Below is an example where counts from taxa present in fewer than 5% of sequence libraries and taxa with less than 0.01% abundance in all sequencing libraries are aggregated together.

$$otu\_filter\left( {micro\_set, \, prev\_cutoff = 5, \, ra\_cutoff = 0.1} \right).$$

### Diversity calculations and analyses

Community-level analyses are an important step for understanding microbial community diversity. The community-level measures summarize key features within individual communities (alpha-diversity) and between individual communities (beta-diversity). As is standard among microbiome pipelines, *tidyMicro* provides optimized algorithms for calculating the following alpha diversity indices from bootstrapped rarefied sequence datasets: ${S}_{obs}$, Chao1, Shannon’s and Simpson’s diversity indices, and Shannon’s and Simpson’s evenness indices. Also included is Good’s coverage estimator, with options to filter out sequencing libraries with low coverage and/or low sequencing depth. *tidyMicro* gives the option to calculate alpha diversity indices for every OTU table or only one specified table. If the study contains an OTU table from multiple environments, each table’s diversity might be useful. Since diversities measure how many different taxa are present and how evenly they are distributed, aggregating counts together into few groups could bias these estimates. For this reason, we recommend only calculating diversity measures on the lowest taxonomic rank if the study has OTU tables of different taxonomic ranks from the same site. Calculating alpha diversities for the family level OTU table is demonstrated below:

$$alpha\_div\left( {micro\_set, \, table = Genus, \, iter = 100, \, min\_depth = 10,000, \, min\_goods = 90} \right).$$

For beta diversity analyses we incorporate the *vegan *[19] package, which provides a large array of methods to generate dissimilarity tables. *vegan* also provides a function to test for differences in beta diversities in a pseudo-regression framework using PERMANOVA.

### Analyses of Individual Taxa

**Fig. 6:**
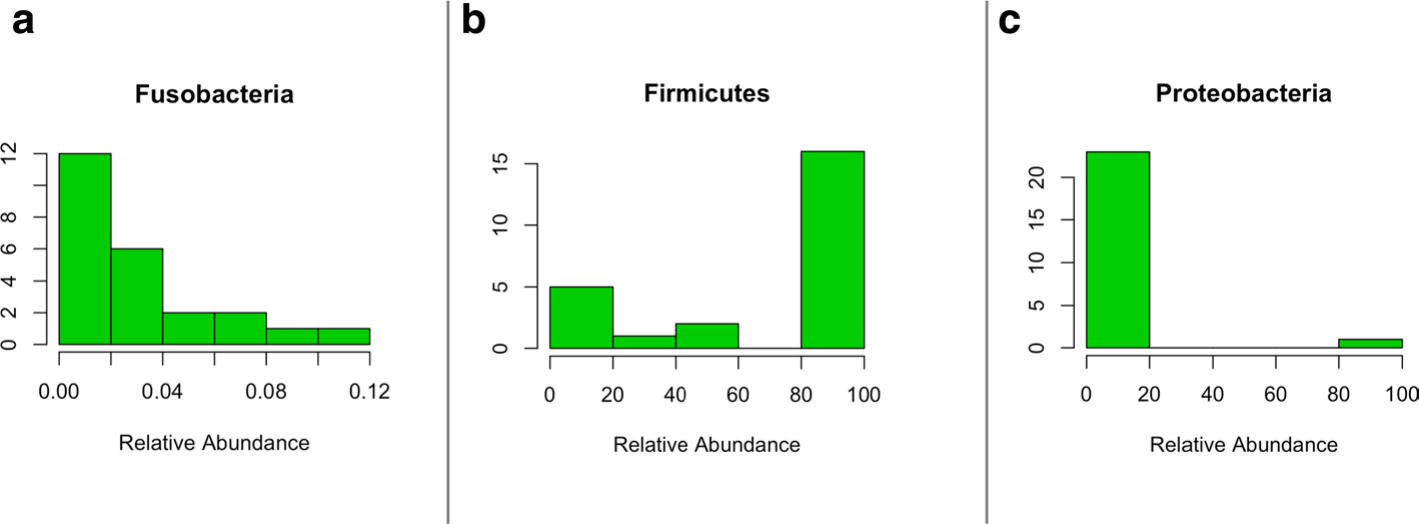
Examples of common taxa abundance distributions. Strong right skews (**a**), “U” shaped distributions (**b**), and sparsity (**c**) are all common patterns

In parallel with community analyses (i.e., alpha- and beta-diversity), identification of individual taxa that differ between groups of subjects often is of interest to investigators. Taxon abundance can be difficult to model due to strong right skews (Fig. 6a), “U” or “J” shapes (Fig. 6b), and sparsity (Fig. 6c). *tidyMicro* provides multiple modeling options and automated model summaries enabling greater user control over their analyses of taxon abundances. This level of flexibility and focus on taxa level models is a novel feature of *tidyMicro*.

Negative binomial and beta binomial regression are the current gold standards for modeling taxon abundance. Each method has advantages and disadvantages. Negative binomial models are often adequate and easier to fit, but they are unable to capture any “U” shapes that may arise in taxa abundances. Beta binomial models offer more flexibility but require more computational time. The negative binomial model coefficients also represent estimated rate ratios, which are a more straightforward representation of associations than the odds ratios provided by beta binomial models. *tidyMicro* has built in functions to fit negative binomial models using the *MASS* package [20] or beta binomial models using the *VGAM* package [21] to every taxa within a specified OTU table. Negative binomial models include sequencing depths as an *offset* term by default to deal with the compositionality of these data, and the beta binomial models handle this by directly modeling the taxa RA. We provide several useful summaries of the models and taxa abundances. An example of fitting negative binomial models to all genus-level taxa with MRSA infection and smoking status as covariates with sequencing depth as an offset term is demonstrated below:

$$nb\_mods\left( {micro\_set, \, table = genus, \, MRSA\_Positive,Smoking,Offset = TRUE} \right).$$

These more complicated parametric models may have convergence issues when applied to microbiome studies and simpler methods may need to be considered. Consequently, *tidyMicro* includes functions to perform rank-sum tests on taxa abundance or Chi-squared tests on taxa presence to address this potential issue. These functions give the user the option to run the test on all taxa or just the taxa for which the regression models failed to converge.

### Model Summaries and Visualizations

Output from (potentially) hundreds of negative binomial or beta binomial models can be difficult to interpret. For this reason, *tidyMicro* includes model summaries and several model-visualization tools. The modeling functions produce summary tables from each taxa’s model (Additional file 1 ) and estimate tables that contain rate ratios (or odds ratios), Wald confidence intervals, and false discovery rate (FDR) adjusted p-values (Additional file 2). When interaction terms are present, the appropriate main effect estimates and covariances are summed together before being exponentiated into rate ratios (or odds ratios) and confidence intervals in the estimate table.

**Fig. 7:**
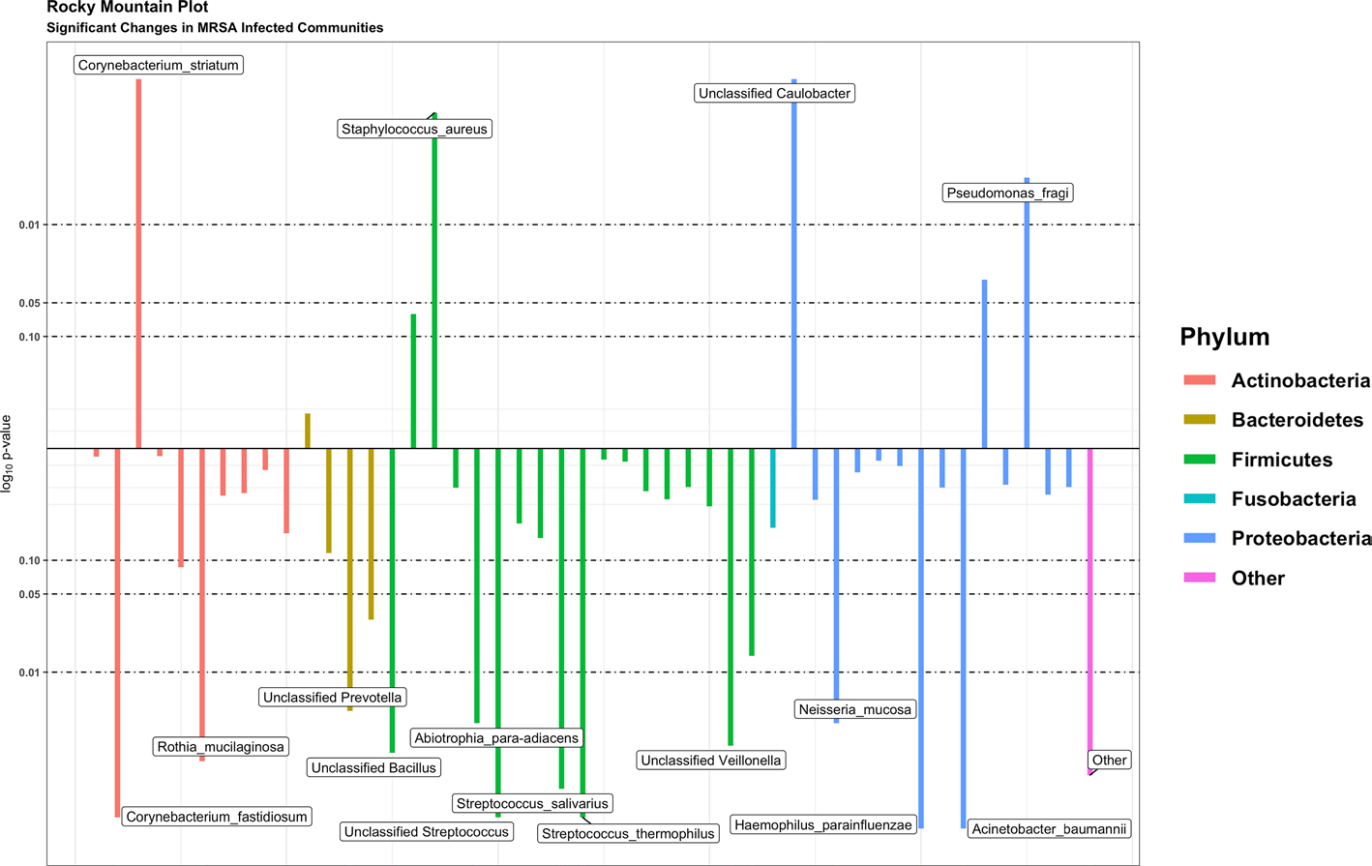
Rocky mountain plot made from negative binomial models. Relationships between MRSA infection and genus level taxa abundance after controlling for smoking status were estimated using negative binomial models using log(sequencing depth) as an offset. All models were fit using the *glm.nb* function in the MASS package. False discovery rate (FDR) adjusted p-values of estimated $\beta$ coefficients are log transformed, and the magnitude is plotted along the y-axis. For positive $\beta$ estimates, the log( FDR p-value) is multiplied by -1, so the direction along the y-axis corresponds to the direction of the estimated relationship

**Fig. 8:**
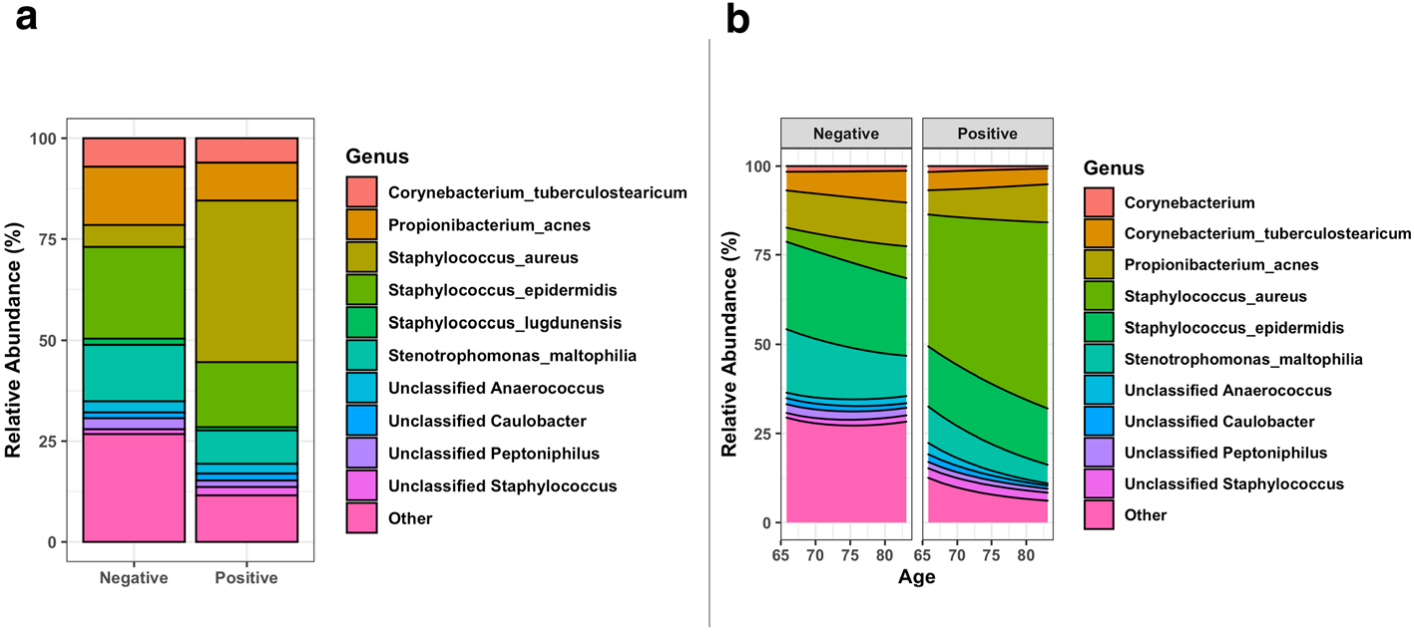
Parametric stacked bar charts. Parametric stacked bar charts back transform $\beta$ parameter estimates to get estimated taxa abundance. (**a**) Parametric stacked bar charts from estimated relationships between MRSA infection and genus level taxa abundances after controlling for smoking status. (**b**) Parametric stacked bar charts from estimated relationships between genus level taxa abundance and subject age by MRSA infection. All models from both (**a**) and (**b**) used log(sequencing depth) as an offset

*tidyMicro* includes heatmaps and forest plots of $\beta$ parameter estimates to help visualize consistent trends. The package also introduces novel regression-based rocky mountain plots and novel parametric stacked bar charts. The regression-based rocky mountain plots are similar to the association based rocky mountain plots used for exploratory data analysis (Fig. 4). FDR p-values of a specified covariate are log transformed and the magnitude is plotted along the y-axis. For positive $\beta$ estimates, the log(FDR p-value) is multiplied by -1 so the direction along the y-axis corresponds to the direction of the estimated relationship (Fig. 7). Parametric stacked bar charts back-transform estimated $\beta$ parameters to estimated taxa abundance. Back-transformed estimates allow the user to plot estimated continuous trends in composition and estimated composition controlling for confounders. Both main effects (Fig. 8a) and interactions (Fig. 8b) can be plotted with the parametric stacked bar charts. An example of plotting the relationship between age and MRSA infection is demonstrated below:

$$nb\_bars(nb\_model,bpd*MRSA\_Positive,top\_taxa = 5).$$

The *top_taxa* argument is used to aggregate the estimated abundances of all but the 5 most abundant into an “Other” category. One can also specify an abundance cutoff for taxa to be excluded from the “Other” category. This functionality exists in all stacked bar chart functions within *tidyMicro*.

## Discussion

As microbiome data and analyses become more common, user accessibility to reliable and extensible analysis pipelines has become increasingly important. Boosting user control through easily understandable and manipulatable data objects is a key step in expanding analysts’ toolkits. The *tidyMicro* pipeline helps meet this need by introducing the principles of tidy data and fully leveraging the *tidyverse* into microbiome analyses. We also extend functionality available in other popular microbiome pipelines with novel exploratory visualization methods (e.g., Rocky Mountain plots, Three Mode PCA and PCoA), a strong focus on modeling of individual taxa abundances with multiple modeling options, and automated summary and estimate tables.

This new tidy data structure paradigm also allows for the extension of *tidyMicro* to other omics data sets. Functions have the generalizability to support count data from any omics data in principle. For example, studies containing multiple omics (genomic, proteomic, metagenomic, marker gene profiles) could combine tables using the *tidy_micro* function as if they were multiple OTU tables and implement Three Mode PCA/PCoA as a data integration technique. Furthermore, as the analysis of microbiome data evolves, other extensions such as alternative modeling (e.g., zero inflated models from DESeq2[22]) and other sequence data formats can be easily included given the flexibility provided in *tidyMicro*.

## Conclusions

*tidyMicro* provides a reliable complement to popular microbiome analysis R packages. We provide standard tools as well as novel extensions on standard analyses to improve interpretability and the analyst’s ability to communicate results while preserving the tidy data format to encourage open source collaboration. This new tidy data structure increases user control over analyses and novel visualizations improve collaborative efforts. External and custom functions can be easily integrated into any workflow. All visualizations are created through the *ggplot2* package in the *tidyverse*[9] providing publication-quality graphics that can be further customized by the use of “geoms.” A full package vignette exploring all functions and function options is available through the package and GitHub. The simple examples and full workflow from the package are reproducible and can serve as a tutorial to analysts new to microbiome data.

**Table 1:** MRSA study cohort demographic information

	*S. aureus* negative (n=26)	*S. aureus* positive (n = 26)	Total
Age	71.81 (± 11.24)	71.67 (± 11.38)	71.74 (± 11.20)
Antibiotics			
No	11 (42.31%)	11 (42.31%)	21 (40.38%)
Yes	15 (57.69%)	15 (57.69%)	31 (59.62%)
Diabetes			
No	13 (50.00%)	13 (50.00%)	26 (50.00%)
Yes	13 (50.00%)	13 (50.00%)	26 (50.00%)
Nasal steroids			
No	23 (88.46%)	23 (88.46%)	46 (88.46%)
Yes	3 (11.54%)	3 (11.54%)	6 (11.54%)
Nursing home			
No	21 (80.77%)	21 (80.77%)	42 (80.77%)
Yes	5 (19.23%)	5 (19.23%)	10 (19.23%)
Smoking			
Never	7 (26.92%)	9 (34.62%)	16 (30.77%)
Former	9 (34.62%)	13 (50.00%)	22 (42.31%)
Current	10 (38.46%)	4 (15.38%)	14 (26.92%)

Continuous variables summarized using means (sd) and discrete variables are summarized using counts (%).

**Table 2:** BPD infant cohort demographic information

	Mild (n = 4)	Moderate (n = 11)	Severe (n = 9)	Total (n = 24)
Birth Weight (Kg)	0.81 (± 0.07)	0.74 (± 0.16)	0.82 (± 0.18)	0.78 (± 0.15)
Sex				
Female	3 (75.00%)	7 (63.64%)	4 (44.44%)	14 (58.33%)
Male	1 (25.00%)	4 (36.36%)	5 (55.56%)	10 (41.67%)
Gestational age (Wks)	25.25 (± 1.50)	25.27 (± 1.10)	25.67 (± 1.22)	25.42 (±1.18)
Maternal ethnicity				
Hispanic	2 (50.00%)	5 (45.45%)	2 (22.22%)	9 (37.50%)
Non-hispanic white	2 (50.00%)	6 (54.55%)	7 (77.78%)	15 (62.50%)

Continuous variables summarized using means (sd) and discrete variables are summarized using counts (%).

## Supplementary Information

**Additional file 1.** Convergent model summary. Column 1: Taxa names. Column 2: Model coefficients. Column 3: β parameter estimates. Column 4: 95% profile likelihood confidence intervals for β estimates. Column 5: Z-statistics. Column 6: P-values from Wald tests on individual beta coefficients. Column 7: False discovery rate adjusted p-values. Column 8: P-values from likelihood ratio test for entire covariate (like an F-test)..

**Additional file 2.** Model estimates table. Column 1: Taxa names. Column 2: Model coefficients. Column 3: Estimated rate ratios from exponentiated β estimates. For models with interaction terms, the appropriate β estimates are summed before being exponentiated. Column 4: Exponentiated 95% Wald confidence intervals. For models with interaction terms, the appropriate β estimates and covariance terms are summed for the Wald intervals. Column 5: Z-statistics from β estimates. Column 6: False discovery rate adjusted p-value

## Data Availability

The datasets generated and/or analyzed during the current study are available in the [data] repository, https://github.com/CharlieCarpenter/tidyMicro. The package and data are also available through the CRAN repository https://cran.r-project.org/web/packages/tidyMicro/index.html

## Abbreviations

OTU: Operational taxonomic unit
MRSA: Methicillin-resistant Staphylococcus aureus
BPD: Bronchopulmonary dysplasia
FDR: False discovery rate
